# Reliability of online dental final exams in the pre and post COVID-19 era: A comparative study

**DOI:** 10.1371/journal.pone.0286148

**Published:** 2023-05-24

**Authors:** Hung Trong Hoang, Phuong Thao Nguyen, Nam Cong-Nhat Huynh, Tam Thi-Thanh Nguyen, Trang Thi Huyen Tu, Michael George Botelho, Lan Van Nguyen, Kaori Shima, Tomonori Sasahira

**Affiliations:** 1 Department of Dental Public Health, Faculty of Odonto-Stomatology, University of Medicine and Pharmacy at Ho Chi Minh City, Ho Chi Minh City, Viet Nam; 2 Department of Molecular Oral Pathology and Oncology, Graduate School of Medical and Dental Science, Kagoshima University, Kagoshima, Kagoshima, Japan; 3 Laboratory of Prosthodontics, Laboratory of Oral-Maxillofacial Biology, Faculty of Odonto-Stomatology, University of Medicine and Pharmacy at Ho Chi Minh City, Ho Chi Minh City, Viet Nam; 4 Department of General Dentistry, Faculty of Odonto-Stomatology, University of Medicine and Pharmacy at Ho Chi Minh City, Ho Chi Minh City, Viet Nam; 5 Department of Dental Basic Sciences, Faculty of Odonto-Stomatology, University of Medicine and Pharmacy at Ho Chi Minh City, Ho Chi Minh City, Viet Nam; 6 Faculty of Dentistry, Prince Philip Dental Hospital, University of Hong Kong, Hong Kong, China; 7 Department of Oral Radiology, Faculty of Odonto-Stomatology, University of Medicine and Pharmacy at Ho Chi Minh City, Ho Chi Minh City, Viet Nam; Sohag University Faculty of Medicine, EGYPT

## Abstract

Amidst the fourth COVID-19 wave in Viet Nam, national lockdowns necessitated the closure of numerous dental schools. To assess DDS (Doctor of Dental Surgery) graduation exams, this study analyzed their 2021 implementation in comparison to onsite exams conducted in 2020 and 2022 at the Faculty of Odonto-Stomatology, University of Medicine and Pharmacy at Ho Chi Minh City, Viet Nam (FOS-UMPH). The final online examination comprises two main sessions: a synchronous online examination using FOS-UMPH e-Learning for theories (consisting of 200 MCQs and 3 written tests with 3 clinical situations needed be solved) and a synchronous online examination using Microsoft Teams for practicum (comprising of 12 online OSCE stations). The final grades were evaluated using the same metrics in face-to-face final examinations in 2022 and 2020. A total of 114, 112 and 95 students were recruited for the first-time exams in 2020, 2021 and 2022, respectively. In order to analyze the reliability, histogram and k-mean clustering were employed. The histograms from 2020, 2021 and 2022 showed a striking similarity. However, fewer students failed in 2021 and 2022 (13% and 12.6%, respectively) compared to 2020 (28%), with clinical problem-solving part grades (belonging to theory session) being notably higher in 2021 and 2022. Intriguingly, the MCQ Score results showed the identical patterns. The courses of orthodontics, dental public health, and pediatrics subjects (in the group of prevention and development dentistry) stood out for their exceptional accuracy across both sessions. After examining data gathered over three years, we identified three distinct clusters: the first comprised of scattered average and low scores, the second characterized by high scores but unstable and scattered and the third cluster boasting consistently high and centered scores. According to our study, online and onsite traditional graduation exam results are relatively equivalent, but additional measures are necessary to standardize the final examination and adapt to the new normal trend in dental education.

## Introduction

The COVID-19 pandemic has had a widespread impact on numerous facets of society, including dental education and assessment of undergraduate students. To cope with the crisis, dental students have had to adapt to a temporary change in their education. Clinical placements have been cancelled, forcing them to switch to remote study from home. Dental schools have responded proactively by introducing online lectures and teaching opportunities to ensure that the students’ learning is not hampered through virtual platforms such as Zoom, Microsoft Teams, sOLAT and Web 2.0 tools [1, 2].

Amid the COVID-19 pandemic, clinicians have admirably balanced their clinical duties with teaching which is highly appreciated. The e-learning course consisted of various components, including educational materials, project tasks, practice tests, and interface ask a teacher, which allowed for a comprehensive online learning experience [2]. However, the limitations of online dental exams include the inability to perform hands-on practical training and the lack of personal interaction between students and patients [3]. Dental students, however, are experiencing some apprehensions regarding the examination process. To mitigate future disruptions, universities are contemplating remote testing as a solution [4–6]. Nevertheless, there are multiple aspects to consider while administering the dental school exam, such as ensuring that students are meeting the learning outcomes and calculating the Educational Performance Measure (EPM). The pandemic has also resulted in the cancellation or postponement of all previously scheduled Objective Structured Clinical Examinations (OSCEs) due to social distancing measurements. Ultimately, learning from predecessors is crucial for students to advance in the field [7]. The absence of clinical experience and skill evaluation has caused has also sparked increasing concerns. Hence, assessing the effectiveness of remote online exams in this regard is imperative.

To objectively uncover novel insights from the data, we employed k-mean clustering analysis in this research. Our study offered an alternative method to assess the reliability of the final exam scores, moving beyond traditional comparative analysis statistics like t-test and one-way ANOVA.

## Materials and methods

### Final excamination procedure

The research plan was approved by the Ethics Committee of University of Medicine and Pharmacy at Ho Chi Minh city. The score collection was approved by “the FOS-UMPH Academic Affairs Unit”.

**Fig 1:**
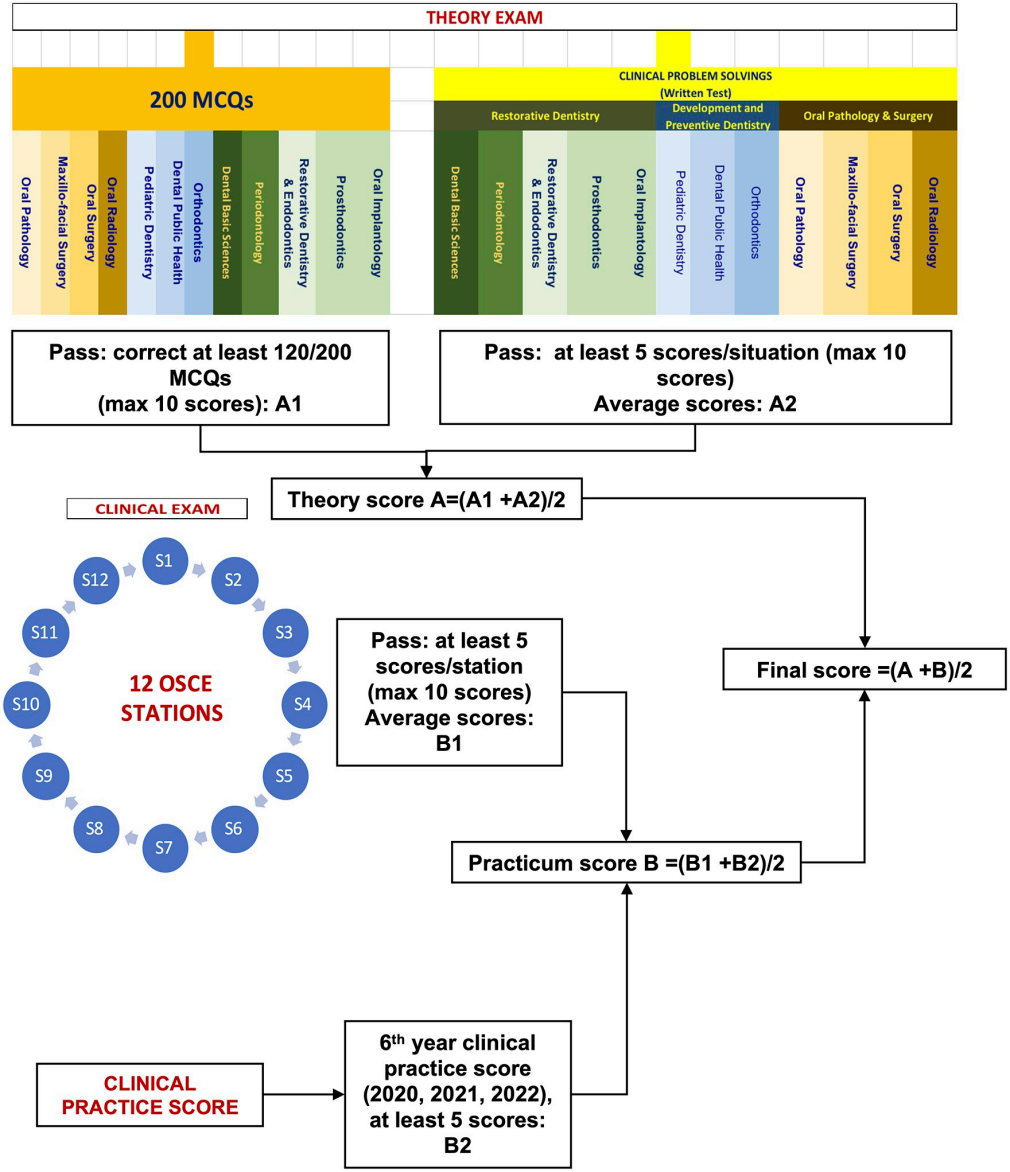
Final exam framework for 6th year dental students. MCQ pass was to correct at least 120/200 MCQs with maximum 10/10 scores. For written test, passing was at least 5 scores/situation (with maximum 10/10 scores). The OSCE pass was at least 5 scores/station (with maximum 10/10 scores). The 6th year clinical practice score was at least 5 scores. Practicum score was average of OSCE and 6th year clinical practice score. Total score was measured as an average of theory and practicum scores.

The final exam framework for 6th year dental students who have been completed their curriculum is detailed in Fig 1. The exam consists of both theory and clinical components. For theory section, students must answer 200 multiple-choices questions (MCQs) spanning 12 disciplines, and complete written tests on three clinical problem-solving scenarios, including Restorative Dentistry, Development and Preventive Dentistry, Oral Pathology and Surgery. To pass the MCQ portion, a minimum of 120/200 questions must be correctly answered with a maximum score of 10/10. In the written test, students must score at least 5 points out of 10 in each scenario to pass. The practical exam is conducted in OSCE format with 12 corresponding to the 12 disciplines. To pass the OSCE, students must at least 5 points out of 10 in each station with a maximum score of 10/10. To ensure a satisfactory evaluation, a minimum score of 5 was required for the 6th year clinical practice. The practicum score was combined average of the OSCE and 6th year clinical practice scores. The total score was determined by averaging of theory and practicum scores.

**Fig 2:**
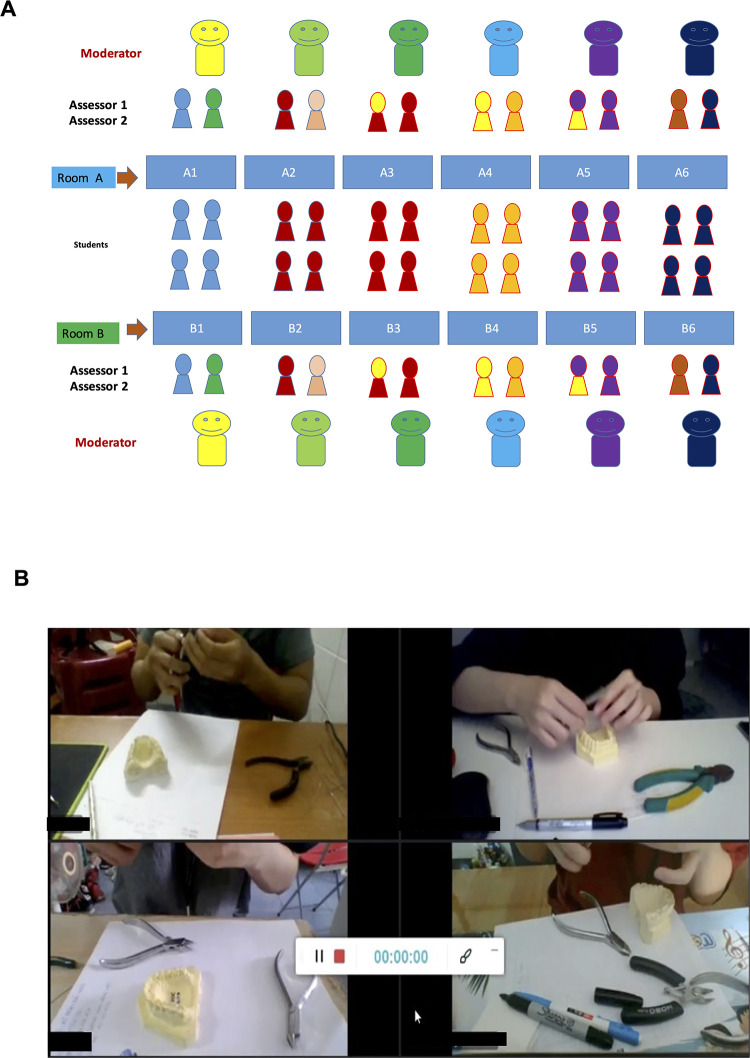
Model of online final exam. **A.** Model for online OSCE Stations: Room A1 (Dental Basic Sciences), A2 (Prosthodontics), A3 (Oral Implantology), A4 (Oral Pathology), A5 (Oral Surgery), A6 (Oral Radiology), B1 (Periodontology), B2 (Restorative Dentistry and Endodontics), B3 (Pediatric Dentistry), B4 (Dental Public Health), B5 (Orthodontics), B6 (Maxillo-facial Surgery) with 2 accessors in each room. **B.** Demonstration of Orthodontics Station. We applied the Team and Channel division functions in Microsoft Teams to assign the students to their rooms after each round of test.

In 2021, conventional assessments comprise in-person theoretical tests, where students are provided with computer system, and clinical examinations where they rotate among different stations and are assessed face-to-face for specific tasks. For virtual assessments, Microsoft Teams (v1.0.23) was utilized to facilitate both theoretical and practical examinations across multiple meeting rooms (Fig 2). We applied the Team and Channel division functions in Microsoft Teams to allocate students to their respective rooms after each round of testing. To ensure a seamless virtual experience, candidates were required to check their internet connectivity, computer compatibility, and availability checklist for the examination. Additionally, virtual waiting rooms were equipped with supervised cameras for both sessions.

### Collection and organization of data

Theory and practicum scores were collected by using the same evaluation metrics in both onsite (2020, 2022) and online (2021). There were 114 students taking the final exam in 2020, 112 students in 2021 and 95 students in 2022 (S1 File). In each data, scores of 12 disciplines of each section (or station) were collected, including:

- Dental Basic Sciences, Periodontology, Restorative Dentistry and Endodontics, Prosthodontics, Oral Implantology (Block of Restorative Dentistry)
- Pediatric Dentistry, Dental Public Health, Orthodontics (Block of Development & Preventive Dentistry)
- Oral Pathology, Maxillo-facial Surgery, Oral Surgery, Oral Radiology (Block of Oral Pathology and Surgery)

### Data observation

Data were processed and analyzed using R version 4.1.3 and RStudio 2021.09.0 Build 351, PBC. We visualized scores in 2020, 2021 and 2022 with histograms, density plots, boxplots using ggplot2 (v3.3.5) and lvplot (v0.2.0) R packages. For each histogram, scores (ranged from 0 to 10 presented by different color) were visualized. For each density plot, scores (ranged from 0 to 10) were visualized with the number of students getting regarding score for 3 groups (2020, 2021 and 2022).

### K-mean clustering analysis

We applied K-mean clustering method by factoextra (v1.0.7), stats (v4.1.1) R packages to analyze score data in three years (S2 File). The package provides convenient functions to extract and visualize the output of multivariate data analyses as our present data, including ’PCA’ (Principal Component Analysis) prior to data visualization by ggplot2 package.

K-means clustering is a method of vector quantization to partition n number of students/ year (observations) into k clusters in which each student (observation) belongs to the cluster with the nearest mean of scores (cluster centers). This results in a partitioning of the data space into Voronoi cells. k-means clustering minimizes within-cluster variances (squared Euclidean distances). At the minimum, all cluster centers were at the mean of their Voronoi sets (the set of data points which were nearest to the cluster center). Algorithm of Hartigan and Wong (1979) was applied by default [8]. Characteristics of each cluster of two datasets were then interpreted and concluded. Firstly, we determined and visualized the optimal number of clusters k (from 1 to 10) in each year. Next, we performed k-means clustering on a data matrix to partition the points of score data into k groups such that the sum of squares from points to the assigned cluster center was minimized. Based on the observation of clusters, we specified k = 3 for the best clarifying (S1 Fig).

### Statistical analysis

All results were expressed as means ± standard error of the mean. Statistical differences were determined by 2-way ANOVA with Bonferroni post-test or by 2-tailed Student’s t test. A *p* value less than 0.05 was considered significant.

## Results

### Distribution of component scores throughout 3 years

**Fig 3:**
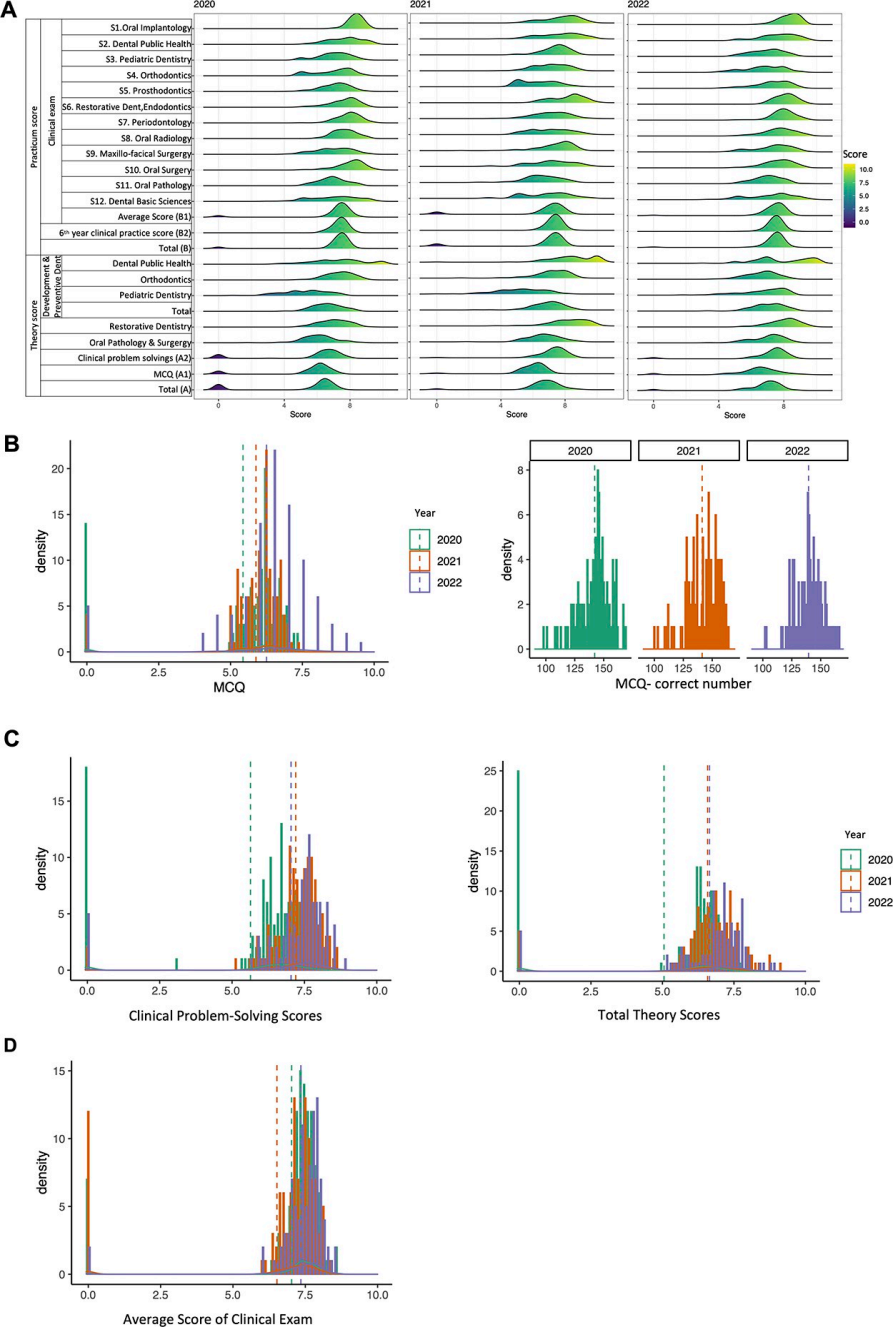
Distribution of scores throughout 3 years. **A.** Component scores of all subjects. **B.** Histograms of MCQ, Total Theory Scores, Clinical Problem-Solving Scores and Average Score of Clinical Exam. For each histogram, scores (ranged from 0 to 10 presented by different color) were visualized. For each density plot, scores (ranged from 0 to 10) were visualized with the number of students getting regarding score for 3 groups (2020, 2021 and 2022). Dash lines presents the mean score of each group.

While the composition score spectrum showed similarity between years in total theory score and total practicum score, there was a disparity in scores between years on closer observation (Fig 3A) (Table 1). In addition, the average scores of each subject were displayed in Table 1, in which, the average practicum scores of clinical subjects (oral implantology, orthodontics, prosthodontics, periodontology, oral radiology, and oral surgery) are significantly different between online and onsite (2020 vs. 2021 and 2021 vs. 2022).

**Table 1:** The difference of average score of each subject in 3 years 2020, 2021 and 2022.

			2020		2021		2022		*p*-value	
			Mean	SE	Mean	SE	Mean	SE	2020 vs. 2021	2021 vs. 2022
Theory score	Development & Preventive Dent	Dental Public Health	7.29	0.15	8.38	0.14	7.85	0.17	***0*.*00***	***0*.*02***
		Orthodontics	7.34	0.08	7.14	0.11	6.64	0.10	*0*.*13*	***0*.*00***
		Pediatric Dentistry	5.18	0.14	5.20	0.14	6.70	0.15	*0*.*90*	***0*.*00***
		Total	6.60	0.08	6.91	0.09	7.06	0.10	***0*.*02***	*0*.*26*
	Restorative Dentistry		7.02	0.09	8.24	0.11	8.00	0.09	***0*.*00***	*0*.*10*
	Oral Pathology & Surgergy		6.10	0.10	6.68	0.10	7.00	0.11	***0*.*00***	***0*.*03***
	Clinical problem solvings (A2)		6.69	0.06	7.33	0.07	7.43	0.07	***0*.*00***	*0*.*31*
	MCQ (A1)		6.19	0.05	6.10	0.05	6.60	0.11	*0*.*24*	***0*.*00***
	MCQ (correct number)		142.02	1.30	141.32	1.39	139.51	1.27	*0*.*64*	*0*.*34*
	**Total (A)**		6.47	0.04	6.89	0.07	7.01	0.08	***0*.*00***	*0*.*24*
Practicum score	Clinical exam	S1.Oral Implantology	8.34	0.04	7.75	0.10	8.31	0.06	***0*.*00***	***0*.*00***
		S2. Dental Public Health	7.68	0.10	7.63	0.12	7.66	0.11	***0*.*04***	*0*.*86*
		S3. Pediatric Dentistry	7.00	0.09	7.41	0.08	6.96	0.10	*0*.*49*	***0*.*00***
		S4. Orthodontics	6.99	0.11	7.09	0.10	6.78	0.13	***0*.*00***	***0*.*04***
		S5. Prosthodontics	7.09	0.08	6.32	0.11	7.26	0.11	***0*.*00***	***0*.*00***
		S6. Restorative Dent, Endodontics	7.62	0.08	8.15	0.09	8.11	0.06	***0*.*00***	*0*.*73*
		S7. Periodontology	7.91	0.07	7.09	0.11	7.95	0.08	***0*.*00***	***0*.*00***
		S8. Oral Radiology	7.65	0.07	7.03	0.12	7.72	0.10	***0*.*05***	***0*.*00***
		S9. Maxillo-facical Surgergy	6.98	0.10	7.39	0.11	7.34	0.12	*0*.*42*	*0*.*74*
		S10. Oral Surgery	7.99	0.08	7.11	0.13	7.64	0.09	***0*.*00***	***0*.*00***
		S11. Oral Pathology	6.92	0.08	6.52	0.10	7.02	0.09	*0*.*39*	***0*.*00***
		S12. Dental Basic Sciences	6.99	0.14	6.79	0.14	7.15	0.15	*0*.*50*	*0*.*07*
		Average Score (B1)	7.49	0.04	7.30	0.05	7.51	0.05	***0*.*02***	***0*.*01***
	6^th^ year clinical practice score (B2)		7.38	0.04	7.35	0.04	7.47	0.05	*0*.*21*	***0*.*03***
	**Total (B)**		7.45	0.03	7.34	0.04	7.49	0.04	***0*.*03***	***0*.*01***

Fig 3B indicated that the distribution of MCQ total score of year 2022 was slightly shifted horizontally along the X-axis and the mean score was higher than 2020 and 2021. In particular, in 2022 examination, there were more students who achieved MCQ score in the range of 7.5–10 than that of 2020 and 2021 examination. In addition, the same tendency in total theory scores of 2022, and there is a relatively small amount of overlap (Fig 3C). Interestingly, regarding to Clinical Problem-Solving scores, the mean score of 2021 was the highest one among the three cohorts. Moreover, the Average Score of Clinical Exam Scores of the 3 years was distributed very similarly (Fig 3D).

### Development and Preventive Dentistry shows the highest homogeneity over the years before, during and after the COVID-2019 pandemic

**Fig 4:**
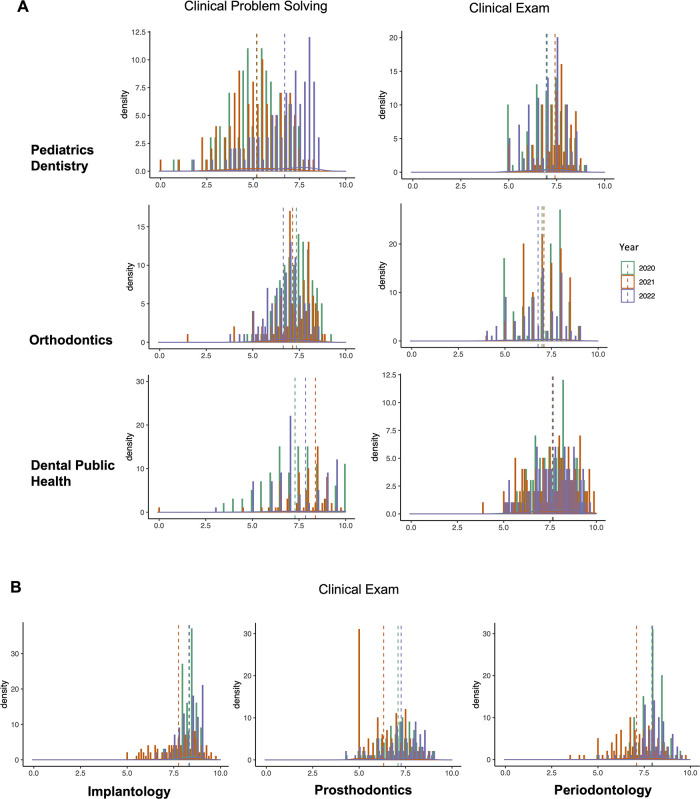
Density plots of scores of Development and Preventive Dentistry in Clinical Problem Solving vs. Clinical Exam throughout 3 years. For each density plot, scores (ranged from 0 to 10) were visualized with the number of students getting regarding score for 3 groups (2020, 2021 and 2022). Dash lines presents the mean score of each group.

Among the final exam subjects, Development and Preventive Dentistry subjects (including Pediatrics Dentistry, Orthodontics, Dental Public Health) had the most overlapped score distribution in both Clinical Problem Solving vs. Clinical Exam forms (Fig 4A) between years (Except the Pediatrics Dentistry score of 2022 in Clinical Problem Solving). Meanwhile, regarding to clinical exam scores of Restorative Dentistry (the block subjects of Periodontology, Prosthodontics, Oral Implantology) in Fig 4B, we observed different distribution shapes, suggesting deviations in students’ performances.

### K-mean clustering analysis revealed 3 identical clusters in each year

**Fig 5:**
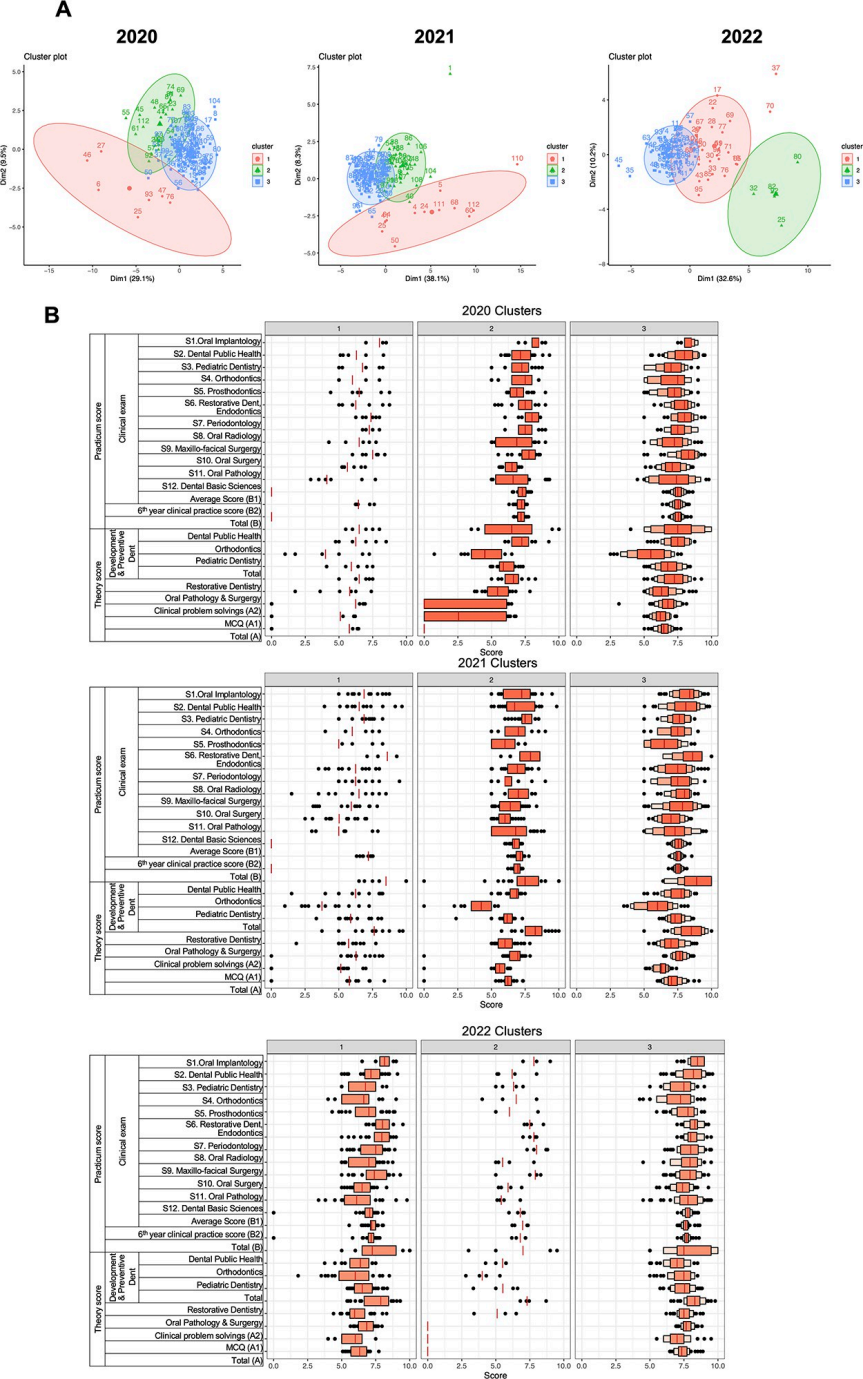
Cluster analysis of final exam scores throughout 3 years. **A.** Principal component analysis (PCA) of scores revealed 3 clusters (red, blue, green colors) in each year. For each year, students were clusters by their score using the 2 first PCA (dimension 1 and 2 with the percentage number presenting the percentage of clarifying of observations). **B.** Boxplot of component scores of all tests in each cluster of each year. Median of score (red lines), outliners (black dots) were ranged from 0 to 10. Cluster 1 (2020), cluster 1 (2021) and cluster 3 (2022) shared the same components. Cluster 2 (2020), cluster 2 (2021) and cluster 2 (2022) shared the same components. Cluster 3 (2020), cluster 3 (2021) and cluster 1 (2022) shared the same components.

To characterize the features of score across 3 years, we performed k-mean clustering analysis. The results revealed 3 clusters in data collected in these 3 years showing the same characteristics (Fig 5A and 5B). The 1st cluster (cluster 2 in 2020, 2021, and 2022) which was scattered average and low scores. The 2nd cluster (cluster 3 in 2020 and 2021, cluster 1 in 2022) which was high scores but not stable and scattered. The 3rd cluster (cluster 1 in 2020 and 2021, cluster 3 in 2022) was high and centered scores. The size of cluster 1, 2 and 3 in 2020 was 85, 7 and 22 students respectively. The size of cluster 1, 2 and 3 in 2021 was 71, 12 and 29 students respectively. The size of cluster 1, 2 and 3 in 2022 was 30, 5 and 60 students respectively. We can see that students in each year were clarified clearly with the compatible sizes for scattered average and low scores groups (7, 12 and 5 students or 6.1%, 10,7% and 5.3% in 2020, 2021 and 2022 respectively), high scores but not stable and scattered groups (22, 29 and 30 students or 19.3%, 25.9% and 31.6% in 2020, 2021 and 2022 respectively), high and centered scores groups (85, 71 and 60 or 74.6%, 63.4% and 63.1% in 2020, 2021 and 2022 respectively).

## Discussion

Viet Nam faced a surge of SARS-COVID19 cases in 2021 that resulted in a public health lockdown from May 31 to September 30. The restrictions imposed included stay-at-home orders, mandatory mask-wearing, social distancing; and gathering limits [9]. The COVID-19 pandemic has brought about new challenges in dental education, and the need for an online evaluation platform has been recognized [10]. It is important to note that online evaluation platforms can be designed in many different ways, and their reliability depend on various factors, such as the number of items, types of questions, and methods used to prevent cheating [11]. Additionally, the reliability of self-reported dental visits may not be as valid as direct clinical examinations, which are considered the gold standard for assessing dental health [12, 13]. In response, our faculty had to organize tele-assessments for final year dental students, with all participants interacting remotely online. The assessment comprised two main sessions: a synchronous online examination for theories and a synchronous online examination for practicum. The grades were evaluated using the same metrics in face-to-face final examination in 2022 and 2020. The findings revealed similarities in the composition score spectrum for theory and practicum scores between years, with disparity noted. Additionally, Development and Preventive Dentistry shows the most consistency over time and K-mean clustering analysis identified three identical clusters in each year.

The initial difference in subject scores between years can be attributed to the variance in assessment tools—online and offline examination. Although online education and evaluation have been around for some time, the recent pandemic has brought it to the forefront. A study by Jagadeesan and Neelakanta (2021) utilized an online self-assessment tool for medical students in biochemistry during the pandemic, highlighting the successful transition from traditional classroom teaching to online education for dentistry as evidenced by examination results [14]. This study contributes to the existing literature by critically examining students’ evaluation performance in higher education during the pandemic online teaching period and suggests that the transition from traditional classroom teaching to online education in dentistry was successful in terms of adaptability as assessed by examination results. The results of the study indicate that utilizing an online model for final exams of dental students in Vietnam is a viable approach. The authenticity and dependability of this method have been established, rendering it apt not just for summative evaluations but also for formative assessments in the dental curriculum, particularly in the digital age and during the era of remote learning.

Online examinations offer several advantages like ease of access, time and cost savings, instant feedback, and multimedia support in question design, among others. They also encourage creative and analytical thinking, provide personalized options for question display, and clarity in answer reception [15, 16]. The study provided valuable insights of online tests. However, online tests may have some limitations as the uncontrollable nature of test takers can compromise the reliability of the test. In our case, due to the Covid19 lockdown, the six-year undergraduate dental program was compelled to shift the final exam to online assessment within a limited timeframe from May 31 to September 30. Insufficient time for clinical practice has negatively impacted students’ Restorative dentistry subject scores in 2021. This particular field demands hundreds of training hours, making it challenging for students to excel without ample practice opportunities. Varied internet speed and connectivity issues may have impacted the exam process, with three cases of disconnection within 10 to 30 seconds. Fortunately, all affected students could reconnect and complete their examinations. Furthermore, each student’s unique examination environment may have influenced their level of focus during the exam.

Despite similarities in course material and lectures, significant differences in practicum exam scores were observed between courses. However, it is important to note that assessment practices are not standardized, and each instructor has their own subject criteria for evaluation. As such, the reliability of our qualitative analysis may be influenced by external factors such as pandemic, adjustments made by teachers to help students perform better, and the challenges posed by transitioning from in-person to online exams, which impact students’ technical, emotional, and intellectual abilities.

The COVID-19 pandemic has led to the closure of many dental schools, thus necessitating the need for effective and reliable online teaching and assessment tools [17]. However, the short amount of time given for online exam preparation due to the pandemic has raised concerns about the reliability of online dental exams. Our study indicates a high reliability, which is similar to other studies [17, 18]. Nevertheless, COVID-19 has limited the value of online oral exams, as they require significant time to ensure fairness and reliability [19, 20].

The consequences of the COVID-19 pandemic might also be a factor that affected the students’ health and psychology in their examination preparation and examination process, but this issue has not been fully investigated in this study. Hence, further research is required to understand the potential challenges and limitations of online dental final exams, and how they may differ from traditional in-person examinations. Additionally, the lack of direct observation and physical interaction with patients during an online exam may limit the ability of examiners to accurately assess a student’s clinical skills and decision-making abilities. These are important considerations that need to be addressed to ensure the reliability of online dental final exams. Further studies are needed to explore these factors in depth and to develop best practices for online dental education and assessment.

## Conclusion

In this study, we applied k-mean clustering analysis as a new approach to evaluate reliability of final exam score besides the conventional statistics based on p-value looking on each single subject. The tool provided a comprehensive vision of the present data with feature discovering of each cluster. On a positive note, our final year written examination and clinical OSCE distance assessments proved successful in evaluating student performance. In light of this, we may consider retaining online written summative MCQs and distance assessments with extended matching questions. However, remote assessment may not be practical for certain OSCE tasks within and beyond our field. While simultaneous remote assessments help evaluate competency with great accuracy, we must prioritize in-person clinical OSCEs whenever feasible.

## Supporting information

S1 Fig WCSS plot (left panel) and heatmap of Euclidian distances (right panel) of 2020 (A), 2021 (B) and 2022 (C). (PDF)

S1 File Full data of 2020, 2021 and 2021 scores. (XLSX)

S2 File R script for analysis and plot generation. (PDF)

## References

[1] O’Doherty D, Dromey M, Lougheed J, Hannigan A, Last J, McGrath D. Barriers and solutions to online learning in medical education–an integrative review. BMC medical education. 2018;18(1):1–11. 29880045 10.1186/s12909-018-1240-0 PMC5992716

[2] Nijakowski K, Lehmann A, Zdrojewski J, Nowak M, Surdacka A. The Effectiveness of the Blended Learning in Conservative Dentistry with Endodontics on the Basis of the Survey among 4th-Year Students during the COVID-19 Pandemic. Int J Environ Res Public Health. 2021;18(9). Epub 2021/05/01. doi: 10.3390/ijerph18094555 . 33923047 PMC8123304

[3] Barabari P, Moharamzadeh K. Novel Coronavirus (COVID-19) and Dentistry-A Comprehensive Review of Literature. Dent J (Basel). 2020;8(2). Epub 2020/05/28. doi: 10.3390/dj8020053 . 32455612 PMC7345990

[4] Appiah M, Van Tonder F. E-Assessment in Higher Education: A Review. International Journal of Business Management & Economic Research. 2018;9(6).

[5] Timmis S, Broadfoot P, Sutherland R, Oldfield A. Rethinking assessment in a digital age: opportunities, challenges and risks. British Educational Research Journal. 2016;42(3):454–76. doi: 10.1002/berj.3215

[6] Durning SJ, Dong T, Ratcliffe T, Schuwirth L, Artino AR Jr., Boulet JR, et al. Comparing Open-Book and Closed-Book Examinations: A Systematic Review. Acad Med. 2016;91(4):583–99. Epub 2015/11/05. doi: 10.1097/ACM.0000000000000977 . 26535862

[7] Ralhan S, Bhogal P, Bhatnagar G, Young J, Green M. Effective teaching skills—how to become a better medical educator. BMJ. 2012;344:e765. doi: 10.1136/bmj.e765

[8] Hartigan JA, Wong MA. Algorithm AS 136: A K-Means Clustering Algorithm. Journal of the Royal Statistical Society Series C (Applied Statistics). 1979;28(1):100–8. doi: 10.2307/2346830

[9] Doan LP, Le Vu MN, Vu GT, Le HT, Nguyen LH, Latkin CA, et al. The COVID-19 endemic in Vietnam: Contextual considerations and implications. Front Public Health. 2023;11:997635. Epub 2023/03/31. doi: 10.3389/fpubh.2023.997635 . 36992873 PMC10040746

[10] Hong S, Go B, Rho J, An S, Lim C, Seo DG, et al. Effects of a blended design of closed-book and open-book examinations on dental students’ anxiety and performance. BMC Med Educ. 2023;23(1):25. Epub 2023/01/13. doi: 10.1186/s12909-023-04014-9 . 36635682 PMC9836918

[11] Porto FR, Ribeiro MA, Ferreira LA, Oliveira RG, Devito KL. In-person and virtual assessment of oral radiology skills and competences by the Objective Structured Clinical Examination. J Dent Educ. 2022. Epub 2022/11/11. doi: 10.1002/jdd.13138 . 36352350

[12] Gilbert GH, Rose JS, Shelton BJ. A prospective study of the validity of data on self-reported dental visits. Community Dent Oral Epidemiol. 2002;30(5):352–62. Epub 2002/09/19. doi: 10.1034/j.1600-0528.2002.00062.x . 12236826

[13] Amavel R, Cruz-Correia R, Frias-Bulhosa J. Remote diagnosis of children dental problems based on non-invasive photographs—a valid proceeding? Stud Health Technol Inform. 2009;150:458–62. Epub 2009/09/12. . 19745354

[14] Jagadeesan AR, Neelakanta RR. Online self-assessment tool in Biochemistry—A medical student’s perception during COVID-19 pandemic. J Educ Health Promot. 2021;10:137. Epub 2021/07/06. doi: 10.4103/jehp.jehp_792_20 . 34222512 PMC8224495

[15] Prigoff J, Hunter M, Nowygrod R. Medical Student Assessment in the Time of COVID-19. J Surg Educ. 2021;78(2):370–4. Epub 2020/08/21. doi: 10.1016/j.jsurg.2020.07.040 . 32819868 PMC7392154

[16] Akram H, Yingxiu Y, Al-Adwan AS, Alkhalifah A. Technology Integration in Higher Education During COVID-19: An Assessment of Online Teaching Competencies Through Technological Pedagogical Content Knowledge Model. Front Psychol. 2021;12:736522. Epub 2021/09/14. doi: 10.3389/fpsyg.2021.736522 . 34512488 PMC8426343

[17] Khalaf K, El-Kishawi M, Moufti MA, Al Kawas S. Introducing a comprehensive high-stake online exam to final-year dental students during the COVID-19 pandemic and evaluation of its effectiveness. Med Educ Online. 2020;25(1):1826861. Epub 2020/10/02. doi: 10.1080/10872981.2020.1826861 . 33000704 PMC7580847

[18] Hakami Z, Khanagar SB, Vishwanathaiah S, Hakami A, Bokhari AM, Jabali AH, et al. Psychological impact of the coronavirus disease 2019 (COVID-19) pandemic on dental students: A nationwide study. J Dent Educ. 2021;85(4):494–503. Epub 2020/11/01. doi: 10.1002/jdd.12470 . 33128397

[19] Elsalem L, Al-Azzam N, Jum’ah AA, Obeidat N. Remote E-exams during Covid-19 pandemic: A cross-sectional study of students’ preferences and academic dishonesty in faculties of medical sciences. Ann Med Surg (Lond). 2021;62:326–33. Epub 2021/02/02. doi: 10.1016/j.amsu.2021.01.054 . 33520225 PMC7825891

[20] Dhahri AA, Arain SY, Memon AM, Rao A, Medical Education Pakistan collaborator g, Mian MA. "The psychological impact of COVID-19 on medical education of final year students in Pakistan: A cross-sectional study". Ann Med Surg (Lond). 2020;60:445–50. Epub 2020/12/01. doi: 10.1016/j.amsu.2020.11.025 . 33251004 PMC7683177

