# Supplementary material for: Reliability of online dental final exams in the pre and post COVID-19 era: A comparative study
Source: PLoS One. 2023 May 24;18(5):e0286148. doi: 10.1371/journal.pone.0286148 (PMC10208487; doi:10.1371/journal.pone.0286148)

Sup FigS1: WCSS plot (left panel) and heatmap of Euclidian distances (right panel) of 2020 (A), 2021 (B) and 2022 (C). WCSS is the sum of the squared distance between each point and the centroid in a cluster.

A

2020

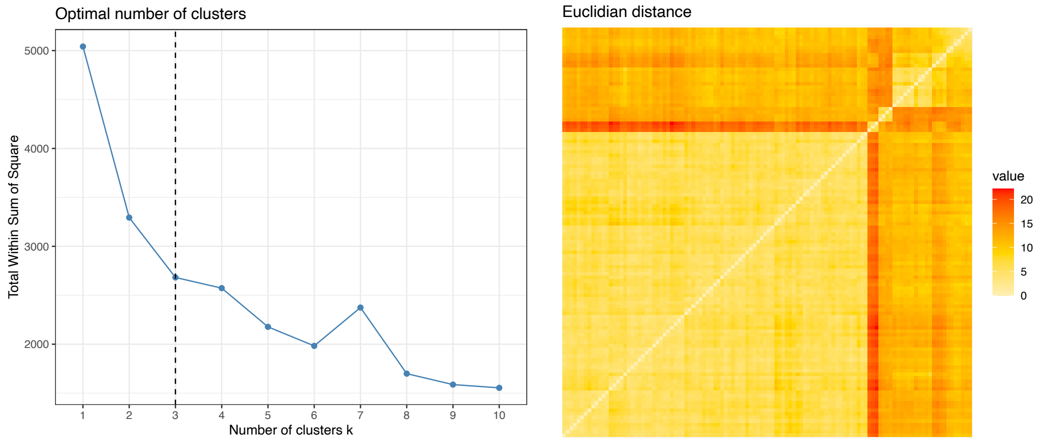

B

2021

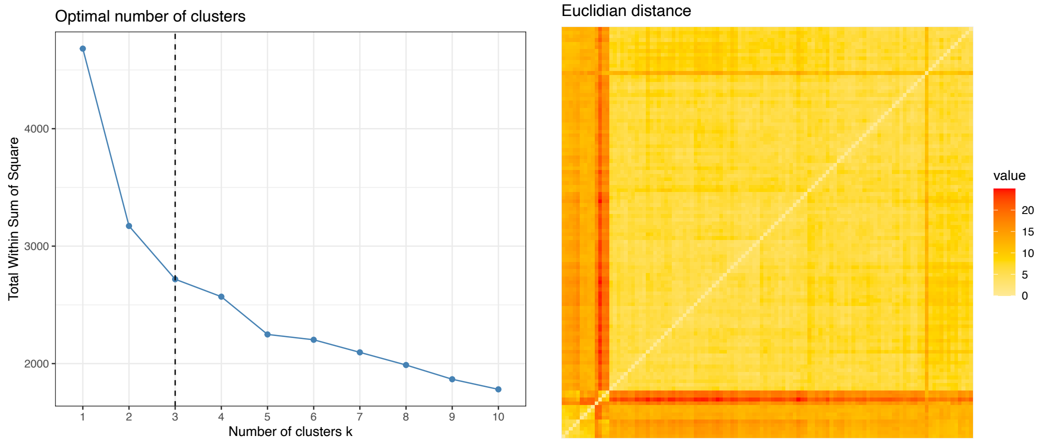

C

2022

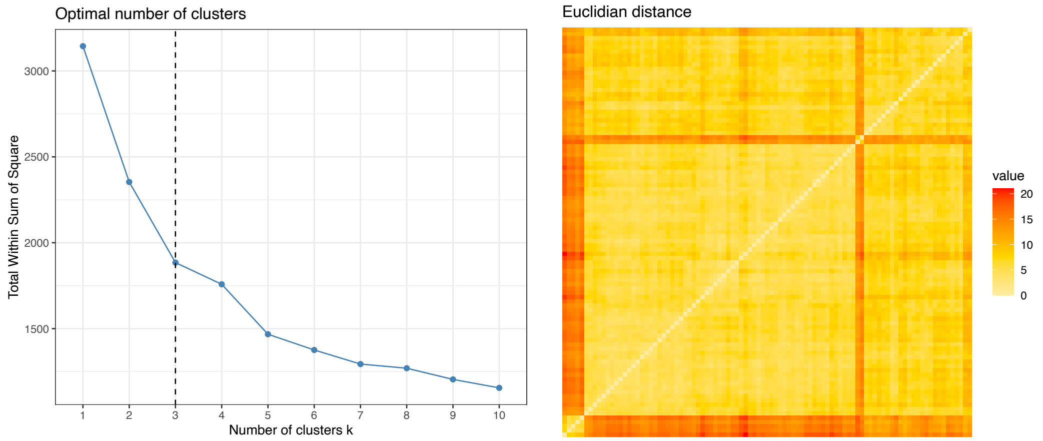

Supplement: S1 Fig — (PDF) [file pone.0286148.s001.pdf]
