# Supplementary material for: Reliability of online dental final exams in the pre and post COVID-19 era: A comparative study
Source: PLoS One. 2023 May 24;18(5):e0286148. doi: 10.1371/journal.pone.0286148 (PMC10208487; doi:10.1371/journal.pone.0286148)
Supplement: S2 File — (PDF) [file pone.0286148.s003.pdf]

```
setwd("~/OneDrive - UMP/RHM/2021-2022/Điểm thi TN online")

library(foreign)
library(tidyverse)
library(ggplot2)
library(RColorBrewer)
library(readxl)
library(plyr)
library(ggridges)
library(viridis)
library(factoextra)
library(lvplot)

# DATA 2020
data_2020 <- read_excel("./Data/Diem thi Tot Nghiep 
2020-2021-2022.xlsx", sheet = 1, skip = 6)
data_2020 <- data_2020[-(115:121),-c(2,9:20)]

data_2020$NAM <- 2020
data_2020$NAM <- as.factor(data_2020$NAM)

colnames(data_2020) <- c('ID', 'NKPH', 'BLPT', 'DPPT.RTE', 
'DPPT.CHRM', 'DPPT.NKCC', 'DPPT.TONG', 'MCQ.CAU', 'MCQ', 'THLS', 
'LTTN',
                         'TTTN.NKCS', 'TTTN.BHM', 'TTTN.PTM', 
'TTTN.PTHM', 'TTTN.CDHA', 'TTTN.NC', 'TTTN.CRNN', 'TTTN.PH',
                         'TTTN.CHRM', 'TTTN.NKTE', 'TTTN.NKCC', 
'TTTN.CGNK', 'THTN.TB', 'THN6', 'THTN', 'NAM')

data_2020[is.na(data_2020)] <- 0

write.table(data_2020, "./Data/data_2020.txt", sep="\t", 
quote=FALSE, row.names=FALSE)


# DATA 2021
data_2021 <- read_excel("./Data/Diem thi Tot Nghiep 
2020-2021-2022.xlsx", sheet = 2, skip = 6)
data_2021 <- data_2021[-c(113:118),-12]

colnames(data_2021) <- c('ID', 'NKPH', 'BLPT', 'DPPT.RTE', 
'DPPT.CHRM', 'DPPT.NKCC', 'DPPT.TONG', 'MCQ.CAU', 'MCQ', 'THLS', 
'CDM','LTTN',
                         'TTTN.NKCS', 'TTTN.BHM', 'TTTN.PTM', 
'TTTN.PTHM', 'TTTN.CDHA', 'TTTN.NC', 'TTTN.CRNN', 'TTTN.PH',
                         'TTTN.CHRM', 'TTTN.NKTE', 'TTTN.NKCC', 
'TTTN.CGNK', 'THTN.TB', 'THN6', 'THTN', 'NAM')

data_2021$NAM <- 2021
data_2021$NAM <- as.factor(data_2021$NAM)

data_2021[is.na(data_2021)] <- 0

write.table(data_2021, "./Data/data_2021.txt", sep="\t", 
```

```
quote=FALSE, row.names=FALSE)

# DATA 2022
data_2022 <- read_excel("./Data/Diem thi Tot Nghiep
2020-2021-2022.xlsx", sheet = 3, skip = 6)
data_2022 <- data_2022[,-27]

colnames(data_2022) <- c('ID', 'NKPH', 'BLPT', 'DPPT.RTE',
'DPPT.CHRM', 'DPPT.NKCC', 'DPPT.TONG', 'MCQ.CAU', 'MCQ',
'THLS','LTTN',
                         'TTTN.NKCS', 'TTTN.BHM', 'TTTN.PTM',
'TTTN.PTHM', 'TTTN.CDHA', 'TTTN.NC', 'TTTN.CRNN', 'TTTN.PH',
                         'TTTN.CHRM', 'TTTN.NKTE', 'TTTN.NKCC',
'TTTN.CGNK', 'THTN.TB', 'THN6', 'THTN', 'NAM')

data_2022$NAM <- 2022
data_2022$NAM <- as.factor(data_2022$NAM)

data_2022[is.na(data_2022)] <- 0

write.table(data_2022, "./Data/data_2022.txt", sep="\t",
quote=FALSE, row.names=FALSE)


pdf("./Output/Fig.1.pdf", useDingbats = F, height = 3, width = 5)

#NKPH
sub.data <- rbind(data_2020[,c(2,27)], data_2021[,c(2,28)],
data_2022[,c(2,27)])
mu <- ddply(sub.data, "NAM", summarise, grp.mean=mean(NKPH))

ggplot(sub.data, aes(NKPH, color = NAM)) +
  xlim(-0.075,10) +
  geom_histogram(binwidth = 0.125, alpha = 0.5, fill="transparent",
position="dodge") +
  geom_vline(data=mu, aes(xintercept=grp.mean, color=NAM),
             linetype="dashed") +
  scale_color_brewer(palette="Dark2") +   geom_density(alpha=0.6) +
  theme_classic()


#BLPT
sub.data <- rbind(data_2020[,c(3,27)], data_2021[,c(3,28)],
data_2022[,c(3,27)])
mu <- ddply(sub.data, "NAM", summarise, grp.mean=mean(BLPT))

ggplot(sub.data, aes(BLPT, color = NAM)) +
  xlim(-0.075,10) +
  geom_histogram(binwidth = 0.125, alpha = 0.5, fill="transparent",
position="dodge") +
  geom_vline(data=mu, aes(xintercept=grp.mean, color=NAM),
             linetype="dashed") +
  scale_color_brewer(palette="Dark2") +   geom_density(alpha=0.6) +
  theme_classic()
```

```
#DPPT.RTE
sub.data <- rbind(data_2020[,c(4,27)], data_2021[,c(4,28)],
data_2022[,c(4,27)])
mu <- ddply(sub.data, "NAM", summarise, grp.mean=mean(DPPT.RTE))

ggplot(sub.data, aes(DPPT.RTE, color = NAM)) +
  xlim(-0.075,10) +
  geom_histogram(binwidth = 0.125, alpha = 0.5, fill="transparent",
position="dodge") +
  geom_vline(data=mu, aes(xintercept=grp.mean, color=NAM),
             linetype="dashed") +
  scale_color_brewer(palette="Dark2") +   geom_density(alpha=0.6) +
  theme_classic()

#DPPT.CHRM
sub.data <- rbind(data_2020[,c(5,27)], data_2021[,c(5,28)],
data_2022[,c(5,27)])
mu <- ddply(sub.data, "NAM", summarise, grp.mean=mean(DPPT.CHRM))

ggplot(sub.data, aes(DPPT.CHRM, color = NAM)) +
  xlim(-0.075,10) +
  geom_histogram(binwidth = 0.125, alpha = 0.5, fill="transparent",
position="dodge") +
  geom_vline(data=mu, aes(xintercept=grp.mean, color=NAM),
             linetype="dashed") +
  scale_color_brewer(palette="Dark2") +   geom_density(alpha=0.6) +
  theme_classic()

#DPPT.NKCC
sub.data <- rbind(data_2020[,c(6,27)], data_2021[,c(6,28)],
data_2022[,c(6,27)])
mu <- ddply(sub.data, "NAM", summarise, grp.mean=mean(DPPT.NKCC))

ggplot(sub.data, aes(DPPT.NKCC, color = NAM)) +
  xlim(-0.075,10) +
  geom_histogram(binwidth = 0.125, alpha = 0.5, fill="transparent",
position="dodge") +
  geom_vline(data=mu, aes(xintercept=grp.mean, color=NAM),
             linetype="dashed") +
  scale_color_brewer(palette="Dark2") +   geom_density(alpha=0.6) +
  theme_classic()

#DPPT.TONG
sub.data <- rbind(data_2020[,c(7,27)], data_2021[,c(7,28)],
data_2022[,c(7,27)])
mu <- ddply(sub.data, "NAM", summarise, grp.mean=mean(DPPT.TONG))

ggplot(sub.data, aes(DPPT.TONG, color = NAM)) +
  xlim(-0.075,10) +
  geom_histogram(binwidth = 0.125, alpha = 0.5, fill="transparent",
position="dodge") +
  geom_vline(data=mu, aes(xintercept=grp.mean, color=NAM),
             linetype="dashed") +
```

```
  scale_color_brewer(palette="Dark2") +   geom_density(alpha=0.6) +
  theme_classic()

#MCQ.CAU
sub.data <- rbind(data_2020[,c(8,27)], data_2021[,c(8,28)],
data_2022[,c(8,27)])
mu <- ddply(sub.data, "NAM", summarise, grp.mean=mean(MCQ.CAU))

ggplot(sub.data, aes(MCQ.CAU, color = NAM)) +
  xlim(90,170) +
  geom_histogram(binwidth = 1, alpha = 0.5, fill="transparent",
position="dodge") +
  geom_vline(data=mu, aes(xintercept=grp.mean, color=NAM),
             linetype="dashed") +
  scale_color_brewer(palette="Dark2") +   geom_density(alpha=0.6) +
  facet_wrap(vars(NAM)) +
  theme_classic()

#MCQ
sub.data <- rbind(data_2020[,c(9,27)], data_2021[,c(9,28)],
data_2022[,c(9,27)])
mu <- ddply(sub.data, "NAM", summarise, grp.mean=mean(MCQ))

ggplot(sub.data, aes(MCQ, color = NAM)) +
  xlim(-0.075,10) +
  geom_histogram(binwidth = 0.125, alpha = 0.5, fill="transparent",
position="dodge") +
  geom_vline(data=mu, aes(xintercept=grp.mean, color=NAM),
             linetype="dashed") +
  scale_color_brewer(palette="Dark2") +   geom_density(alpha=0.6) +
  theme_classic()

#THLS
sub.data <- rbind(data_2020[,c(10,27)], data_2021[,c(10,28)],
data_2022[,c(10,27)])
mu <- ddply(sub.data, "NAM", summarise, grp.mean=mean(THLS))

ggplot(sub.data, aes(THLS, color = NAM)) +
  xlim(-0.075,10) +
  geom_histogram(binwidth = 0.125, alpha = 0.5, fill="transparent",
position="dodge") +
  geom_vline(data=mu, aes(xintercept=grp.mean, color=NAM),
             linetype="dashed") +
  scale_color_brewer(palette="Dark2") +   geom_density(alpha=0.6) +
  theme_classic()

#LTTN
sub.data <- rbind(data_2020[,c(11,27)], data_2021[,c(12,28)],
data_2022[,c(11,27)])
mu <- ddply(sub.data, "NAM", summarise, grp.mean=mean(LTTN))

ggplot(sub.data, aes(LTTN, color = NAM)) +
  xlim(-0.075,10) +
  geom_histogram(binwidth = 0.125, alpha = 0.5, fill="transparent",
```

```
position="dodge") +
  geom_vline(data=mu, aes(xintercept=grp.mean, color=NAM),
             linetype="dashed") +
  scale_color_brewer(palette="Dark2") +   geom_density(alpha=0.6) +
  theme_classic()

#TTTN.NKCS
sub.data <- rbind(data_2020[,c(12,27)], data_2021[,c(13,28)],
data_2022[,c(12,27)])
mu <- ddply(sub.data, "NAM", summarise, grp.mean=mean(TTTN.NKCS))

ggplot(sub.data, aes(TTTN.NKCS, color = NAM)) +
  xlim(-0.075,10) +
  geom_histogram(binwidth = 0.125, alpha = 0.5, fill="transparent",
position="dodge") +
  geom_vline(data=mu, aes(xintercept=grp.mean, color=NAM),
             linetype="dashed") +
  scale_color_brewer(palette="Dark2") +   geom_density(alpha=0.6) +
  theme_classic()

#TTTN.BHM
sub.data <- rbind(data_2020[,c(13,27)], data_2021[,c(14,28)],
data_2022[,c(13,27)])
mu <- ddply(sub.data, "NAM", summarise, grp.mean=mean(TTTN.BHM))

ggplot(sub.data, aes(TTTN.BHM, color = NAM)) +
  xlim(-0.075,10) +
  geom_histogram(binwidth = 0.125, alpha = 0.5, fill="transparent",
position="dodge") +
  geom_vline(data=mu, aes(xintercept=grp.mean, color=NAM),
             linetype="dashed") +
  scale_color_brewer(palette="Dark2") +   geom_density(alpha=0.6) +
  theme_classic()

#TTTN.PTM
sub.data <- rbind(data_2020[,c(14,27)], data_2021[,c(15,28)],
data_2022[,c(14,27)])
mu <- ddply(sub.data, "NAM", summarise, grp.mean=mean(TTTN.PTM))

ggplot(sub.data, aes(TTTN.PTM, color = NAM)) +
  xlim(-0.075,10) +
  geom_histogram(binwidth = 0.125, alpha = 0.5, fill="transparent",
position="dodge") +
  geom_vline(data=mu, aes(xintercept=grp.mean, color=NAM),
             linetype="dashed") +
  scale_color_brewer(palette="Dark2") +   geom_density(alpha=0.6) +
  theme_classic()

#TTTN.PTHM
sub.data <- rbind(data_2020[,c(15,27)], data_2021[,c(16,28)],
data_2022[,c(15,27)])
mu <- ddply(sub.data, "NAM", summarise, grp.mean=mean(TTTN.PTHM))

ggplot(sub.data, aes(TTTN.PTHM, color = NAM)) +
```

```
  xlim(-0.075,10) +
  geom_histogram(binwidth = 0.125, alpha = 0.5, fill="transparent",
position="dodge") +
  geom_vline(data=mu, aes(xintercept=grp.mean, color=NAM),
             linetype="dashed") +
  scale_color_brewer(palette="Dark2") +   geom_density(alpha=0.6) +
  theme_classic()

#TTTN.CDHA
sub.data <- rbind(data_2020[,c(16,27)], data_2021[,c(17,28)],
data_2022[,c(16,27)])
mu <- ddply(sub.data, "NAM", summarise, grp.mean=mean(TTTN.CDHA))

ggplot(sub.data, aes(TTTN.CDHA, color = NAM)) +
  xlim(-0.075,10) +
  geom_histogram(binwidth = 0.125, alpha = 0.5, fill="transparent",
position="dodge") +
  geom_vline(data=mu, aes(xintercept=grp.mean, color=NAM),
             linetype="dashed") +
  scale_color_brewer(palette="Dark2") +   geom_density(alpha=0.6) +
  theme_classic()

#TTTN.NC
sub.data <- rbind(data_2020[,c(17,27)], data_2021[,c(18,28)],
data_2022[,c(17,27)])
mu <- ddply(sub.data, "NAM", summarise, grp.mean=mean(TTTN.NC))

ggplot(sub.data, aes(TTTN.NC, color = NAM)) +
  xlim(-0.075,10) +
  geom_histogram(binwidth = 0.125, alpha = 0.5, fill="transparent",
position="dodge") +
  geom_vline(data=mu, aes(xintercept=grp.mean, color=NAM),
             linetype="dashed") +
  scale_color_brewer(palette="Dark2") +   geom_density(alpha=0.6) +
  theme_classic()

#TTTN.CRNN
sub.data <- rbind(data_2020[,c(18,27)], data_2021[,c(19,28)],
data_2022[,c(18,27)])
mu <- ddply(sub.data, "NAM", summarise, grp.mean=mean(TTTN.CRNN))

ggplot(sub.data, aes(TTTN.CRNN, color = NAM)) +
  xlim(-0.075,10) +
  geom_histogram(binwidth = 0.125, alpha = 0.5, fill="transparent",
position="dodge") +
  geom_vline(data=mu, aes(xintercept=grp.mean, color=NAM),
             linetype="dashed") +
  scale_color_brewer(palette="Dark2") +   geom_density(alpha=0.6) +
  theme_classic()

#TTTN.PH
sub.data <- rbind(data_2020[,c(19,27)], data_2021[,c(20,28)],
data_2022[,c(19,27)])
mu <- ddply(sub.data, "NAM", summarise, grp.mean=mean(TTTN.PH))
```

```
ggplot(sub.data, aes(TTTN.PH, color = NAM)) +
  xlim(-0.075,10) +
  geom_histogram(binwidth = 0.125, alpha = 0.5, fill="transparent",
position="dodge") +
  geom_vline(data=mu, aes(xintercept=grp.mean, color=NAM),
             linetype="dashed") +
  scale_color_brewer(palette="Dark2") +   geom_density(alpha=0.6) +
  theme_classic()

#TTTN.CHRM
sub.data <- rbind(data_2020[,c(20,27)], data_2021[,c(21,28)],
data_2022[,c(20,27)])
mu <- ddply(sub.data, "NAM", summarise, grp.mean=mean(TTTN.CHRM))

ggplot(sub.data, aes(TTTN.CHRM, color = NAM)) +
  xlim(-0.075,10) +
  geom_histogram(binwidth = 0.125, alpha = 0.5, fill="transparent",
position="dodge") +
  geom_vline(data=mu, aes(xintercept=grp.mean, color=NAM),
             linetype="dashed") +
  scale_color_brewer(palette="Dark2") +   geom_density(alpha=0.6) +
  theme_classic()

#TTTN.NKTE
sub.data <- rbind(data_2020[,c(21,27)], data_2021[,c(22,28)],
data_2022[,c(21,27)])
mu <- ddply(sub.data, "NAM", summarise, grp.mean=mean(TTTN.NKTE))

ggplot(sub.data, aes(TTTN.NKTE, color = NAM)) +
  xlim(-0.075,10) +
  geom_histogram(binwidth = 0.125, alpha = 0.5, fill="transparent",
position="dodge") +
  geom_vline(data=mu, aes(xintercept=grp.mean, color=NAM),
             linetype="dashed") +
  scale_color_brewer(palette="Dark2") +   geom_density(alpha=0.6) +
  theme_classic()

#TTTN.NKCC
sub.data <- rbind(data_2020[,c(22,27)], data_2021[,c(23,28)],
data_2022[,c(22,27)])
mu <- ddply(sub.data, "NAM", summarise, grp.mean=mean(TTTN.NKCC))

ggplot(sub.data, aes(TTTN.NKCC, color = NAM)) +
  xlim(-0.075,10) +
  geom_histogram(binwidth = 0.125, alpha = 0.5, fill="transparent",
position="dodge") +
  geom_vline(data=mu, aes(xintercept=grp.mean, color=NAM),
             linetype="dashed") +
  scale_color_brewer(palette="Dark2") +   geom_density(alpha=0.6) +
  theme_classic()

#TTTN.CGNK
sub.data <- rbind(data_2020[,c(23,27)], data_2021[,c(24,28)],
```

```
data_2022[,c(23,27)])
mu <- ddply(sub.data, "NAM", summarise, grp.mean=mean(TTTN.CGNK))

ggplot(sub.data, aes(TTTN.CGNK, color = NAM)) +
  xlim(-0.075,10) +
  geom_histogram(binwidth = 0.125, alpha = 0.5, fill="transparent",
position="dodge") +
  geom_vline(data=mu, aes(xintercept=grp.mean, color=NAM),
             linetype="dashed") +
  scale_color_brewer(palette="Dark2") +   geom_density(alpha=0.6) +
  theme_classic()

#THTN.TB
sub.data <- rbind(data_2020[,c(24,27)], data_2021[,c(25,28)],
data_2022[,c(24,27)])
mu <- ddply(sub.data, "NAM", summarise, grp.mean=mean(THTN.TB))

ggplot(sub.data, aes(THTN.TB, color = NAM)) +
  xlim(-0.075,10) +
  geom_histogram(binwidth = 0.125, alpha = 0.5, fill="transparent",
position="dodge") +
  geom_vline(data=mu, aes(xintercept=grp.mean, color=NAM),
             linetype="dashed") +
  scale_color_brewer(palette="Dark2") +   geom_density(alpha=0.6) +
  theme_classic()

#THN6
sub.data <- rbind(data_2020[,c(25,27)], data_2021[,c(26,28)],
data_2022[,c(25,27)])
mu <- ddply(sub.data, "NAM", summarise, grp.mean=mean(THN6))

ggplot(sub.data, aes(THN6, color = NAM)) +
  xlim(-0.075,10) +
  geom_histogram(binwidth = 0.125, alpha = 0.5, fill="transparent",
position="dodge") +
  geom_vline(data=mu, aes(xintercept=grp.mean, color=NAM),
             linetype="dashed") +
  scale_color_brewer(palette="Dark2") +   geom_density(alpha=0.6) +
  theme_classic()

#THTN
sub.data <- rbind(data_2020[,c(26,27)], data_2021[,c(27,28)],
data_2022[,c(26,27)])
mu <- ddply(sub.data, "NAM", summarise, grp.mean=mean(THTN))

ggplot(sub.data, aes(THTN, color = NAM)) +
  xlim(-0.075,10) +
  geom_histogram(binwidth = 0.125, alpha = 0.5, fill="transparent",
position="dodge") +
  geom_vline(data=mu, aes(xintercept=grp.mean, color=NAM),
             linetype="dashed") +
  scale_color_brewer(palette="Dark2") +   geom_density(alpha=0.6) +
  theme_classic()
```

```
dev.off()

#Arrange
data_2020b <- data_2020[,-c(8, 27)]
data_2020c <- data_2020b %>% gather("Exam", "Score", -ID)

data_2021b <- data_2021[,-c(8,11, 28)]
data_2021c <- data_2021b %>% gather("Exam", "Score", -ID)

data_2022b <- data_2022[,-c(8, 27)]
data_2022c <- data_2022b %>% gather("Exam", "Score", -ID)

#Fig2
pdf("./Output/Fig.2.pdf", useDingbats = F, height = 8, width = 6)
data_2020c%>%
  mutate(Exam = factor(Exam, levels=c("LTTN", "MCQ", "THLS", "BLPT",
"NKPH", "DPPT.TONG", "DPPT.RTE", 'DPPT.CHRM',
'DPPT.NKCC','THTN','THN6', 'THTN.TB',
                                     'TTTN.NKCS', 'TTTN.BHM',
'TTTN.PTM', 'TTTN.PTHM', 'TTTN.CDHA', 'TTTN.NC', 'TTTN.CRNN',
'TTTN.PH',
                                     'TTTN.CHRM', 'TTTN.NKTE',
'TTTN.NKCC', 'TTTN.CGNK'))) %>%
  ggplot()+
  geom_density_ridges_gradient(aes(x=Score,y=Exam,fill=..x..),
                               scale=1,
                               show.legend = T)+
  scale_fill_viridis(option="D")+
  labs(y="Exams")+
  theme_bw()+ggtitle("2020")

data_2021c%>%
  mutate(Exam = factor(Exam, levels=c("LTTN", "MCQ", "THLS", "BLPT",
"NKPH", "DPPT.TONG", "DPPT.RTE", 'DPPT.CHRM',
'DPPT.NKCC','THTN','THN6', 'THTN.TB',
                                     'TTTN.NKCS', 'TTTN.BHM',
'TTTN.PTM', 'TTTN.PTHM', 'TTTN.CDHA', 'TTTN.NC', 'TTTN.CRNN',
'TTTN.PH',
                                     'TTTN.CHRM', 'TTTN.NKTE',
'TTTN.NKCC', 'TTTN.CGNK'))) %>%
  ggplot()+
  geom_density_ridges_gradient(aes(x=Score,y=Exam,fill=..x..),
                               scale=1,
                               show.legend = T)+
  scale_fill_viridis(option="D")+
  labs(y="Exams")+
  theme_bw()+ggtitle("2021")

data_2022c%>%
  mutate(Exam = factor(Exam, levels=c("LTTN", "MCQ", "THLS", "BLPT",
"NKPH", "DPPT.TONG", "DPPT.RTE", 'DPPT.CHRM',
'DPPT.NKCC','THTN','THN6', 'THTN.TB',
                                     'TTTN.NKCS', 'TTTN.BHM',
'TTTN.PTM', 'TTTN.PTHM', 'TTTN.CDHA', 'TTTN.NC', 'TTTN.CRNN',
```

```
'TTTN.PH',
                                    'TTTN.CHRM', 'TTTN.NKTE',
'TTTN.NKCC', 'TTTN.CGNK'))) %>%
  ggplot()+
  geom_density_ridges_gradient(aes(x=Score,y=Exam,fill=..x..),
                               scale=1,
                               show.legend = T)+
  scale_fill_viridis(option="D")+
  labs(y="Exams")+
  theme_bw()+ggtitle("2022")

dev.off()

# Clustering 2020
data_2020d <- data_2020b[,-1]
pdf("./Output/Fig.3_2020.pdf", useDingbats = F, height = 5, width =
6)
fviz_nbclust(data_2020d, kmeans, method = "wss") +
  theme_bw()+
  geom_vline(xintercept =3, linetype = 2)

km.res <- kmeans(data_2020d, 3, nstart = 25)

fviz_cluster(km.res,
             data=data_2020d,
             ellipse.type = "t",
             ggtheme = theme_classic()
)

# Abnormal highest distance
data_2020d%>%get_dist(method = "euclidean")%>%
  fviz_dist(gradient = list(low = "white", mid = "gold", high =
"red"))+
  scale_x_discrete(labels = NULL,breaks=NULL)+
  scale_y_discrete(labels = NULL,breaks=NULL)+
  coord_equal()+
  ggtitle("Euclidian distance")

data_2020d%>%get_dist(method = "manhattan")%>%
  fviz_dist(gradient = list(low = "white", mid = "pink", high =
"purple"))+
  scale_x_discrete(labels = NULL,breaks=NULL)+
  scale_y_discrete(labels = NULL,breaks=NULL)+
  coord_equal()+
  ggtitle("Manhattan distance")

# Top 10 outliers
centers <- km.res$centers[km.res$cluster, ]
distances <- sqrt(rowSums((data_2020d - centers)^2))
outliers <- order(distances, decreasing=T)[1:10]
plot_df<-mutate(data_2020d,cluster=factor(km.res$cluster))
out_df<-plot_df%>%.[outliers,]

ggplot()+
```

```
  geom_point(data=plot_df,aes(x=MCQ,y=THLS,col=cluster),alpha=0.5)+

geom_point(data=as.data.frame(km.res$centers),aes(x=MCQ,y=THLS,col=f
actor(c(1:3))),shape=15,size=3)+

geom_point(data=out_df,aes(x=MCQ,y=THLS),shape=21,size=6,stroke=1,co
l="red",alpha=0.9)+
  theme_bw()

ggplot()+
  geom_point(data=plot_df,aes(x=LTTN,y=THLS,col=cluster),alpha=0.5)+

geom_point(data=as.data.frame(km.res$centers),aes(x=LTTN,y=THLS,col=
factor(c(1:3))),shape=15,size=3)+

geom_point(data=out_df,aes(x=LTTN,y=THLS),shape=21,size=6,stroke=1,c
ol="red",alpha=0.9)+
  theme_bw()

ggplot()+
  geom_point(data=plot_df,aes(x=NKPH,y=THLS,col=cluster),alpha=0.5)+

geom_point(data=as.data.frame(km.res$centers),aes(x=NKPH,y=THLS,col=
factor(c(1:3))),shape=15,size=3)+

geom_point(data=out_df,aes(x=NKPH,y=THLS),shape=21,size=6,stroke=1,c
ol="red",alpha=0.9)+
  theme_bw()

ggplot()+
  geom_point(data=plot_df,aes(x=LTTN,y=THTN,col=cluster),alpha=0.5)+

geom_point(data=as.data.frame(km.res$centers),aes(x=LTTN,y=THTN,col=
factor(c(1:3))),shape=15,size=3)+

geom_point(data=out_df,aes(x=LTTN,y=THTN),shape=21,size=6,stroke=1,c
ol="red",alpha=0.9)+
  theme_bw()

dev.off()

pdf("./Output/Fig.4_2020.pdf", useDingbats = F, height = 6, width =
9)
data_2020d %>% mutate(cluster=factor(km.res$cluster))%>%
  gather(NKPH:THTN,key="Exam",value="Score")%>%
  mutate(Exam = factor(Exam, levels=c("LTTN", "MCQ", "THLS", "BLPT",
"NKPH", "DPPT.TONG", "DPPT.RTE", 'DPPT.CHRM',
'DPPT.NKCC','THTN','THN6', 'THTN.TB',
                                      'TTTN.NKCS', 'TTTN.BHM',
'TTTN.PTM', 'TTTN.PTHM', 'TTTN.CDHA', 'TTTN.NC', 'TTTN.CRNN',
'TTTN.PH',
                                      'TTTN.CHRM', 'TTTN.NKTE',
'TTTN.NKCC', 'TTTN.CGNK'))) %>%
  ggplot()+
```

```
  geom_lv(aes(x=Exam,y=Score,fill=..LV..),col="black",show.legend =
F,)+
  facet_wrap(~cluster,ncol=3)+
  coord_flip()+
  scale_fill_brewer(palette="Reds",direction = -1)+
  theme_bw()

dev.off()

getmode <- function(v) {
  uniqv <- unique(v)
  uniqv[which.max(tabulate(match(v, uniqv)))]
}
mode <- aggregate(data_2020d, by=list(cluster=km.res$cluster),
getmode)
write.table(mode, "./Output/Mode_of_clusters_2020.txt", sep="\t",
quote=FALSE, row.names=FALSE)

mean <- aggregate(data_2020d, by=list(cluster=km.res$cluster), mean)
write.table(mean, "./Output/Mean_of_clusters_2020.txt", sep="\t",
quote=FALSE, row.names=FALSE)

# Clustering 2021
data_2021d <- data_2021b[,-1]
pdf("./Output/Fig.3_2021.pdf", useDingbats = F, height = 5, width =
6)
fviz_nbclust(data_2021d, kmeans, method = "wss") +
  theme_bw()+
  geom_vline(xintercept =3, linetype = 2)

km.res <- kmeans(data_2021d, 3, nstart = 25)

fviz_cluster(km.res,
             data=data_2021d,
             ellipse.type = "t",
             ggtheme = theme_classic()
)

# Abnormal highest distance
data_2021d%>%get_dist(method = "euclidean")%>%
  fviz_dist(gradient = list(low = "white", mid = "gold", high =
"red"))+
  scale_x_discrete(labels = NULL,breaks=NULL)+
  scale_y_discrete(labels = NULL,breaks=NULL)+
  coord_equal()+
  ggtitle("Euclidian distance")

data_2021d%>%get_dist(method = "manhattan")%>%
  fviz_dist(gradient = list(low = "white", mid = "pink", high =
"purple"))+
  scale_x_discrete(labels = NULL,breaks=NULL)+
  scale_y_discrete(labels = NULL,breaks=NULL)+
  coord_equal()+
  ggtitle("Manhattan distance")
```

```
# Top 10 outliers
centers <- km.res$centers[km.res$cluster, ]
distances <- sqrt(rowSums((data_2021d - centers)^2))
outliers <- order(distances, decreasing=T)[1:10]
plot_df<-mutate(data_2021d,cluster=factor(km.res$cluster))
out_df<-plot_df%>%.[outliers,]

ggplot()+
  geom_point(data=plot_df,aes(x=MCQ,y=THLS,col=cluster),alpha=0.5)+

geom_point(data=as.data.frame(km.res$centers),aes(x=MCQ,y=THLS,col=f
actor(c(1:3))),shape=15,size=3)+

geom_point(data=out_df,aes(x=MCQ,y=THLS),shape=21,size=6,stroke=1,co
l="red",alpha=0.9)+
  theme_bw()

ggplot()+
  geom_point(data=plot_df,aes(x=LTTN,y=THLS,col=cluster),alpha=0.5)+

geom_point(data=as.data.frame(km.res$centers),aes(x=LTTN,y=THLS,col=
factor(c(1:3))),shape=15,size=3)+

geom_point(data=out_df,aes(x=LTTN,y=THLS),shape=21,size=6,stroke=1,c
ol="red",alpha=0.9)+
  theme_bw()

ggplot()+
  geom_point(data=plot_df,aes(x=NKPH,y=THLS,col=cluster),alpha=0.5)+

geom_point(data=as.data.frame(km.res$centers),aes(x=NKPH,y=THLS,col=
factor(c(1:3))),shape=15,size=3)+

geom_point(data=out_df,aes(x=NKPH,y=THLS),shape=21,size=6,stroke=1,c
ol="red",alpha=0.9)+
  theme_bw()

ggplot()+
  geom_point(data=plot_df,aes(x=LTTN,y=THTN,col=cluster),alpha=0.5)+

geom_point(data=as.data.frame(km.res$centers),aes(x=LTTN,y=THTN,col=
factor(c(1:3))),shape=15,size=3)+

geom_point(data=out_df,aes(x=LTTN,y=THTN),shape=21,size=6,stroke=1,c
ol="red",alpha=0.9)+
  theme_bw()

dev.off()

pdf("./Output/Fig.4_2021.pdf", useDingbats = F, height = 6, width =
9)
data_2021d %>% mutate(cluster=factor(km.res$cluster))%>%
  gather(NKPH:THTN,key="Exam",value="Score")%>%
```

```
  mutate(Exam = factor(Exam, levels=c("LTTN", "MCQ", "THLS", "BLPT",
"NKPH", "DPPT.TONG", "DPPT.RTE", 'DPPT.CHRM',
'DPPT.NKCC','THTN','THN6', 'THTN.TB',
                                      'TTTN.NKCS', 'TTTN.BHM',
'TTTN.PTM', 'TTTN.PTHM', 'TTTN.CDHA', 'TTTN.NC', 'TTTN.CRNN',
'TTTN.PH',
                                      'TTTN.CHRM', 'TTTN.NKTE',
'TTTN.NKCC', 'TTTN.CGNK'))) %>%
  ggplot()+
  geom_lv(aes(x=Exam,y=Score,fill=..LV..),col="black",show.legend =
F,)+
  facet_wrap(~cluster,ncol=3)+
  coord_flip()+
  scale_fill_brewer(palette="Reds",direction = -1)+
  theme_bw()

dev.off()

getmode <- function(v) {
  uniqv <- unique(v)
  uniqv[which.max(tabulate(match(v, uniqv)))]
}
mode <- aggregate(data_2021d, by=list(cluster=km.res$cluster),
getmode)
write.table(mode, "./Output/Mode_of_clusters_2021.txt", sep="\t",
quote=FALSE, row.names=FALSE)

mean <- aggregate(data_2021d, by=list(cluster=km.res$cluster), mean)
write.table(mean, "./Output/Mean_of_clusters_2021.txt", sep="\t",
quote=FALSE, row.names=FALSE)

# Clustering 2022
data_2022d <- data_2022b[,-1]
pdf("./Output/Fig.3_2022.pdf", useDingbats = F, height = 5, width =
6)
fviz_nbclust(data_2022d, kmeans, method = "wss") +
  theme_bw()+
  geom_vline(xintercept =3, linetype = 2)

km.res <- kmeans(data_2022d, 3, nstart = 25)

fviz_cluster(km.res,
             data=data_2022d,
             ellipse.type = "t",
             ggtheme = theme_classic()
)

# Abnormal highest distance
data_2022d%>%get_dist(method = "euclidean")%>%
  fviz_dist(gradient = list(low = "white", mid = "gold", high =
"red"))+
  scale_x_discrete(labels = NULL,breaks=NULL)+
  scale_y_discrete(labels = NULL,breaks=NULL)+
  coord_equal()+
```

```
  ggtitle("Euclidian distance")

data_2022d%>%get_dist(method = "manhattan")%>%
  fviz_dist(gradient = list(low = "white", mid = "pink", high =
"purple"))+
  scale_x_discrete(labels = NULL,breaks=NULL)+
  scale_y_discrete(labels = NULL,breaks=NULL)+
  coord_equal()+
  ggtitle("Manhattan distance")

# Top 10 outliers
centers <- km.res$centers[km.res$cluster, ]
distances <- sqrt(rowSums((data_2022d - centers)^2))
outliers <- order(distances, decreasing=T)[1:10]
plot_df<-mutate(data_2022d,cluster=factor(km.res$cluster))
out_df<-plot_df%>%.[outliers,]

ggplot()+
  geom_point(data=plot_df,aes(x=MCQ,y=THLS,col=cluster),alpha=0.5)+

geom_point(data=as.data.frame(km.res$centers),aes(x=MCQ,y=THLS,col=f
actor(c(1:3))),shape=15,size=3)+

geom_point(data=out_df,aes(x=MCQ,y=THLS),shape=21,size=6,stroke=1,co
l="red",alpha=0.9)+
  theme_bw()

ggplot()+
  geom_point(data=plot_df,aes(x=LTTN,y=THLS,col=cluster),alpha=0.5)+

geom_point(data=as.data.frame(km.res$centers),aes(x=LTTN,y=THLS,col=
factor(c(1:3))),shape=15,size=3)+

geom_point(data=out_df,aes(x=LTTN,y=THLS),shape=21,size=6,stroke=1,c
ol="red",alpha=0.9)+
  theme_bw()

ggplot()+
  geom_point(data=plot_df,aes(x=NKPH,y=THLS,col=cluster),alpha=0.5)+

geom_point(data=as.data.frame(km.res$centers),aes(x=NKPH,y=THLS,col=
factor(c(1:3))),shape=15,size=3)+

geom_point(data=out_df,aes(x=NKPH,y=THLS),shape=21,size=6,stroke=1,c
ol="red",alpha=0.9)+
  theme_bw()

ggplot()+
  geom_point(data=plot_df,aes(x=LTTN,y=THTN,col=cluster),alpha=0.5)+

geom_point(data=as.data.frame(km.res$centers),aes(x=LTTN,y=THTN,col=
factor(c(1:3))),shape=15,size=3)+

geom_point(data=out_df,aes(x=LTTN,y=THTN),shape=21,size=6,stroke=1,c
```

```
ol="red",alpha=0.9)+
  theme_bw()

dev.off()

pdf("./Output/Fig.4_2022.pdf", useDingbats = F, height = 6, width =
9)
data_2022d %>% mutate(cluster=factor(km.res$cluster))%>%
  gather(NKPH:THTN,key="Exam",value="Score")%>%
  mutate(Exam = factor(Exam, levels=c("LTTN", "MCQ", "THLS", "BLPT",
"NKPH", "DPPT.TONG", "DPPT.RTE", 'DPPT.CHRM',
'DPPT.NKCC','THTN','THN6', 'THTN.TB',
                                      'TTTN.NKCS', 'TTTN.BHM',
'TTTN.PTM', 'TTTN.PTHM', 'TTTN.CDHA', 'TTTN.NC', 'TTTN.CRNN',
'TTTN.PH',
                                      'TTTN.CHRM', 'TTTN.NKTE',
'TTTN.NKCC', 'TTTN.CGNK'))) %>%
  ggplot()+
  geom_lv(aes(x=Exam,y=Score,fill=..LV..),col="black",show.legend =
F,)+
  facet_wrap(~cluster,ncol=3)+
  coord_flip()+
  scale_fill_brewer(palette="Reds",direction = -1)+
  theme_bw()

dev.off()

getmode <- function(v) {
  uniqv <- unique(v)
  uniqv[which.max(tabulate(match(v, uniqv)))]
}
mode <- aggregate(data_2022d, by=list(cluster=km.res$cluster),
getmode)
write.table(mode, "./Output/Mode_of_clusters_2022.txt", sep="\t",
quote=FALSE, row.names=FALSE)

mean <- aggregate(data_2022d, by=list(cluster=km.res$cluster), mean)
write.table(mean, "./Output/Mean_of_clusters_2022.txt", sep="\t",
quote=FALSE, row.names=FALSE)
```
